# Early Life Microcirculatory Plasticity and Blood Pressure Changes in Low Birth Weight Infants Born to Normotensive Mothers: A Cohort Study

**DOI:** 10.1093/ajh/hpz034

**Published:** 2019-03-01

**Authors:** Muti Goloba, Rajendra Raghuraman, Nansi Botros, Uzma Khan, Monique Klein, Amelia Brown, Donovan Duffy, Nick Anim-Nyame, Duolao Wang, Isaac Manyonda, Tarek F Antonios

**Affiliations:** 1 Molecular and Clinical Sciences Research Institute, St George’s, University of London, London, UK; 2 Blood Pressure Unit, St George’s University Hospitals NHS Foundation Trust, London, UK; 3 Neonatal Unit, St George’s University Hospitals NHS Foundation Trust, London, UK; 4 Obstetrics and Gynaecology Department, Kingston Hospital NHS Foundation Trust, London, UK; 5 Department of Clinical Sciences, Liverpool School of Tropical Medicine, Liverpool, UK; 6 Obstetrics and Gynaecology Department, St George’s University Hospitals NHS Foundation Trust, London, UK

**Keywords:** blood pressure, capillary rarefaction, essential hypertension, hypertension, low birth weight, microcirculation

## Abstract

**BACKGROUND:**

Capillary rarefaction (CR) is an established hallmark of essential hypertension (EH). The aim of this study was to examine early changes in capillary density (CD) and blood pressure (BP) in low birth weight (LBW) infants who are at risk of developing EH in later life.

**METHODS:**

We studied 77 LBW infants and 284 normal birth weight (NBW) infants, all born to mothers with normotension, in a longitudinal multicenter study. Intravital capillaroscopy was used to measure functional basal capillary density (BCD) and maximal capillary density (MCD) at birth, 3, 6, and 12 months.

**RESULTS:**

We found that LBW infants, born preterm and at term, had a significantly higher CD at birth, then underwent significant CR in the 1st 3 months culminating in a CD similar to that seen in NBW infants. NBW infants showed a gradual reduction in CD between birth and 12 months. Non-Caucasian ethnicity and preterm birth were significant predictors of a higher CD at birth. Systolic BP in NBW infants increased significantly from birth to 3 months, and we identified a significant negative correlation between systolic BP and MCD.

**CONCLUSIONS:**

This study has identified a process of early “accelerated capillary remodeling” in LBW infants, which corrects their higher CD at birth. This remodeling is unlikely to explain the CR seen in adult individuals with, or at risk of developing EH. Further follow-up studies are required to determine the timing and mechanisms involved in CR, which is likely to occur after the 1st year of life but before early adulthood.

Essential hypertension (EH) is a major risk factor for cardiovascular disease^1^ but its cause(s) remains elusive. Individuals with EH are known to have significant structural and functional microcirculatory abnormalities when compared with normotensives. Capillary rarefaction (CR), defined as a reduction in spatial capillary density (CD), is an established hallmark of EH and is evident before the development of the disease.^2–5^ The consistent finding of CR in adult individuals with normotension destined to develop hypertension,^5,6^ as well as in borderline hypertensive cohorts,^3^ invites this pertinent question: how early in life does pathological CR occur and could such knowledge provide pointers as to how the growing EH pandemic could be prevented?

A strong body of evidence indicates that low birth weight (LBW) <2.5 kg is associated with vascular abnormalities, the development of hypertension, stroke, and increased mortality in adult life.^7–9^ The mechanism(s) for these associations is not known. We have previously reported that LBW infants born at term or preterm to mothers with normotension do not have CR: instead, these infants have a significantly higher CD at birth compared with their normal birth weight (NBW) counterparts.^10^ Our findings appeared to contrast with reports that functional and structural microcirculatory abnormalities, including CR, are already present in childhood and young adulthood, respectively, in individuals born with LBW.^11–16^ We, therefore, conducted a longitudinal study of a large cohort of infants from birth over a 1-year period.

## METHODS

G-CROS is a British Heart Foundation-funded, prospective cohort study that was approved by the National Research Ethics Service—Dulwich (14/LO/0690). Recruitment was carried out at St George’s University Hospitals NHS Foundation Trust and Kingston Hospital NHS Foundation Trust, London, UK. Infants were recruited across all birth weight categories, gestations, and ethnicities over a 33-month period between August 2014 and April 2017 following written informed parental consent. Each infant was studied on 4 occasions: at birth, 3, 6, and 12 months. Both preterm (i.e., born at <37 weeks’ gestation) and term (i.e., born at ≥37 weeks’ gestation) were included. All participants across all study groups were given a 1-month window either side of their expected follow-up visit to schedule an appointment. The follow-up time points were therefore 3, 6, and 12 months (± 1 month at each stage). The control group was defined as NBW infants born at term. Infants born to mothers with hypertension and/or type 1 diabetes mellitus were retrospectively excluded.

### Intravital capillaroscopy

Two independent observers, M.G. and R.R., used the CapiScope Handheld Video Capillaroscopy System (KK Technology, Devon, England, UK) to measure the CD on the plantar surface of each infant’s big toe. Four microscopic fields were recorded continuously for 30 seconds. Basal CD (BCD), which represents functional CD, was calculated as the mean of these 4 microscopic fields. We then used venous congestion to maximize the number of visualized perfused skin *capillaries*^10,17,18^ by applying a size-appropriate neonatal blood pressure (BP) cuff around the calf muscle. The cuff was then inflated and maintained at 30 mm Hg for 2 minutes to determine the maximal CD (MCD), which represents the structural (anatomical) CD.^17^ BCD was counted on the running video and all capillaries were counted; including continuously and intermittently perfused capillaries. MCD was counted on stills of the video during venous congestion. Skin and room temperatures were monitored during the study using Tele-Thermometers (YSI Inc., Dayton, OH). The total number of capillaries was counted offline using the CapiScope 4.35.0.0 computer software (KK-Technology, Exeter, UK).

### BP measurement

We used the Welch Allyn VSM 300 monitor to measure the infants’ BP in a temperature-controlled room (22–24°C). A neonatal and appropriately sized BP cuff was used on the calf with the infant in a resting state.

### Demographic and maternal factors

We collected maternal details including ethnicity, maternal age, body mass index, BP, smoking, and alcohol history. We measured postnatal growth by measuring the infants’ birth weight (BW), head circumference, body length, mid-arm circumference, and foot width and length. The birth centile was manually plotted for each infant on a “UK-WHO growth chart 0–4 years”. A BW below or equal to the 10th centile was classified as small for gestational age (SGA). Appropriate for gestational age (AGA) was defined as a BW above the 10th centile.

### Intra- and interobserver reliability analysis

The reliability of CD measurements was assessed by estimating the intraclass correlation coefficient (ICC) for the intra- and interobserver reliability, between 2 independent observers: M.G. and R.R. The intraobserver reliability of our method was found to be moderate for MCD measurement (ICC = 0.73) and good for BCD measurement (ICC = 0.86). The interobserver reliability of our method was found to be excellent for both MCD and BCD measurements (ICC = 0.90 and ICC = 0.97, respectively).

### Statistical analysis

The primary end points were defined as the BCD and MCD at the following time points: 3, 6, and 12 months. Between-group and within-group differences in BCD and MCD were analyzed using a mixed model in which BW category/prematurity (preterm vs. term), measurement time point, and interaction between BW category/prematurity and measurement time point were treated as fixed effects, and the subject was treated as a random effect. In addition, mixed models with time-varying covariates were employed to assess the effects of these covariates on the BCD, MCD, percentage change in BCD and percentage change in MCD. The estimated between-group and within-group differences from the mixed models are reported together with their 95% confidence intervals. Correlation coefficients between BP and MCD at different time points were also calculated. Reported *P* values are 2-sided and a *P* value of <0.05 was considered statistically significant. All statistical analyses were carried out by using the Statistical Analysis System, version 9.3 (SAS Institute, Inc., Cary, NC).

## RESULTS

Table 1 shows the baseline characteristics of the study cohort. We included a total of 361 infants in this study; 77 with LBW and 284 NBW infants. NBW infants showed a gradual reduction in BCD and MCD in the 1st 12 months of life. The greatest reduction in BCD occurred between birth and 3 months (mean difference [MD]= –27.62 cap/field, 95% CI [–31.22 to –24.01], *P* < 0.0001), followed by a smaller reduction between 3 and 6 months (MD = –12.64 cap/field, 95% CI [–17.19 to –8.09], *P* < 0.0001). Similarly, the greatest reduction in MCD occurred between birth and 3 months (MD = –31.49 cap/field, 95% CI [–35.63 to –27.34], *P* < 0.0001). Although BCD continued to reduce after 6 months, MCD did not show a significant reduction between 6 and 12 months (MD = –3.29 cap/field, 95% CI [–8.22 to 1.64], *P* = 0.1902; Table 2).

**Table 1.** Baseline characteristics of study cohort

	NBW (*n* = 284)	LBW (*n* = 77)
Maternal demographics		
Age, (years)	33.2 ± 4.6	33.47 ± 5.9
BMI at booking	24.5 ± 4.6	24.1 ± 4.1
Booking SBP (mm Hg)	113.0 ± 10.5	108.9 ± 11.7
Booking DBP (mm Hg)	66.8 ± 7.3	65.4 ± 12.8
Smoking during pregnancy	11 (3.9)	2 (2.6)
Gestational diabetes	22 (7.7)	5 (6.5)
Infant demographics		
Gestation at birth (weeks)	39.4 ± 1.3	35.9 ± 2.0
Preterm birth (< 37 weeks)	0 (0)	46 (59.7)
Term birth (≥ 37 weeks)	284 (100)	31 (40.3)
Sex of baby		
Male	150 (52.8)	38 (49.4)
Female	134 (47.1)	39 (50.6)
Ethnicity		
Caucasian	272 (95.7)	55 (71.4)
Non-Caucasian	11 (3.9)	22 (28.6)
Birth weight of baby (g)	3431.9 ± 468.8	2225.5 ± 205.1

Data are mean ± SD or *n* (%)

BMI, body mass index; DBP, diastolic blood pressure; LBW, low birth weight; NBW, normal birth weight; SBP, systolic blood pressure; SD, standard deviation.

**Table 2.** Mixed model analysis of basal and maximal capillary density and blood pressures in normal birth weight and low birth weight infants: within-group comparison

Variable	Mean (SD)	Within-group comparisons	Mean difference [95% CI]	*P* value
Normal birth weight infants				
Basal capillary density				
Birth	97.44 (19.10)			
3 months	69.83 (9.20)	Birth vs. 3 months	–27.62 [–31.22 to –24.01]*	<0.0001
6 months	56.74 (8.28)	6 vs. 3 months	–12.64 [–17.19 to –8.09]*	<0.0001
12 months	50.81 (9.41)	12 vs. 6 months	–6.53 [–11.16 to –1.91]^†^	0.0058
Maximal capillary density				
Birth	104.23 (16.39)			
3 months	72.76 (11.51)	Birth vs. 3 months	–31.49 [–35.63 to –27.34]*	<0.0001
6 months	57.71 (10.23)	6 vs. 3 months	–14.09 [–19.22 to –8.95]*	<0.0001
12 months	55.33 (11.07)	12 vs. 6 months	–3.29 [–8.22 to 1.64]	0.1902
Systolic BP				
Birth	73.77 (10.67)			
3 months	85.47 (15.43)	3 months vs. birth	12.23 [6.35 to 18.11]*	0.0001
6 months	88.06 (12.65)	6 vs. 3 months	2.42 [–6.53 to 11.37]	0.590
12 months	88.56 (17.11)	12 vs. 6 months	–0.42 [–9.47 to 8.62]	0.926
Diastolic BP				
Birth	46.62 (10.24)			
3 months	49.56 (13.14)	3 months vs. birth	3.84 [–1.75 to 9.43]	0.174
6 months	53.70 (11.28)	6 vs. 3 months	3.13 [–5.38 to 11.64]	0.463
12 months	57.91 (16.27)	12 vs. 6 months	4.33 [–4.17 to 12.83]	0.311
Low birth weight infants				
Basal capillary density				
Birth	111.74 (19.10)			
3 months	64.42 (10.90)	Birth vs. 3 months	–47.01 [–54.33 to –39.69]*	<0.0001
6 months	59.15 (8.71)	6 vs. 3 months	–5.79 [–14.66 to 3.07]	0.1991
12 months	53.20 (9.35)	12 vs. 6 months	–6.18 [–14.47 to 2.12]	0.1435
Maximal capillary density				
Birth	117.12 (19.22)			
3 months	67.40 (12.15)	Birth vs. 3 months	–48.01 [–55.83 to –40.19]*	<0.0001
6 months	62.78 (10.88)	6 vs. 3 months	–6.42 [–16.15 to 3.32]	0.1949
12 months	57.41 (11.77)	12 vs. 6 months	–5.42 [–14.69 to 3.85]	0.2506
Systolic BP				
Birth	76.44 (11.38)			
3 months	80.92 (10.92)	3 months vs. birth	3.50 [–6.60 to 13.60]	0.489
6 months	82.00 (18.22)	6 vs. 3 months	1.35 [–10.88 to 13.58]	0.826
12 months	88.62 (15.86)	12 vs. 6 months	6.85 [–4.71 to 18.40]	0.239
Diastolic BP				
Birth	50.77 (10.92)			
3 months	48.33 (16.33)	3 months vs. birth	–2.79 [–12.40 to 6.81]	0.561
6 months	48.00 (13.71)	6 vs. 3 months	–0.62 [–12.25 to 11.01]	0.915
12 months	54.13 (14.98)	12 vs. 6 months	7.05 [–3.94 to 18.04]	0.204

Within-group differences in BCD and MCD were analysed using a mixed model in which BW category, measurement time point, and interaction between BW category and measurement time point were treated as fixed effects, and the subject was treated as a random effect. BP, blood pressure; BCD, basal capillary density; CI, confidence intervals; MCD, maximal capillary density; SD, standard deviation.

*Significant at *P* < 0.0001.

^†^Significant at *P* < 0.01.

Table 2 shows the within-group comparisons conducted in the LBW cohort. LBW infants showed a significant reduction in BCD between birth and 3 months (MD = –47.01 cap/field, 95% CI [–54.33 to –39.69], *P* <0.0001) Similarly, a significant reduction in MCD was found during this same period of time (mean difference = –48.01 cap/field, 95% CI [–55.83 to –40.19], *P* < 0.0001). Although the NBW cohort demonstrated further smaller reductions in BCD and MCD in the period between 3 and 6 months; the LBW cohort did not show any further significant reductions in CD after 3 months.

### LBW vs. NBW infants

**Figure 1.**
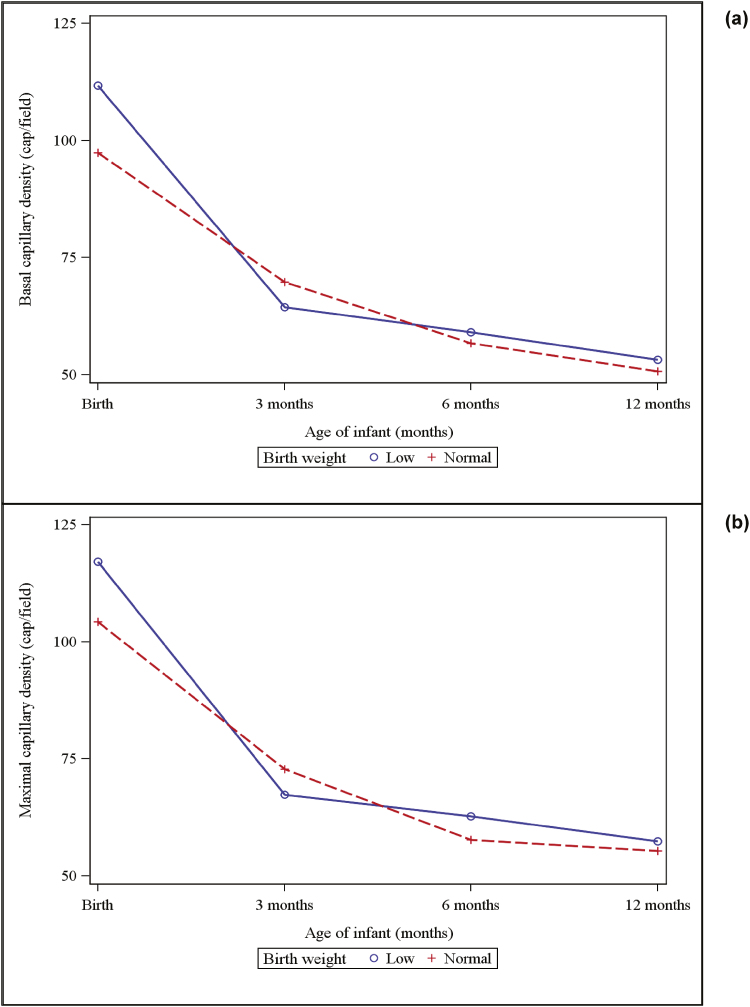
Capillary density in low birth weight and normal birth weight infants at birth, 3 months, 6 months and 12 months. (**a**) Basal (functional) capillary density (**b**) maximal (structural) capillary density.

Table 3 shows the results of comparisons between NBW and LBW cohorts. LBW infants had a significantly higher BCD and MCD at birth in of all gestations, when compared with the NBW group (BCD MD = 14.32 cap/field, 95% CI [10.73 to 17.90], *P* < 0.0001 and MCD MD = 12.83, 95% CI [8.77 to 16.88], *P* < 0.0001). LBW infants also demonstrated a significantly higher percentage reduction in BCD (MD = 7.81%, 95% CI [1.30 to 14.32], *P* = 0.0194) and MCD (MD = 8.29%, 95% CI [0.56 to 16.02], *P* = 0.0361) between birth and 3 months when compared to the NBW group (Figure 1).

**Table 3.** Mixed model analysis of capillary density and blood pressures at birth, 3, 6, and 12 months in normal birth weight and low birth weight infants: between-group comparison

Variable	NBW Mean (SD)	LBW Mean (SD)	Mean difference [95% CI] LBW vs. NBW	*P* value
Basal capillary density				
Birth	97.44 (19.10)	111.74 (19.10)	14.32 [10.73 to 17.90]*	<0.0001
3 months	69.83 (9.20)	64.42 (10.90)	–5.08 [–12.64 to 2.49]	0.1876
6 months	56.74 (8.28)	59.15 (8.71)	1.78 [–5.27 to 8.82]	0.6199
12 months	50.81 (9.41)	53.20 (9.35)	2.13 [–4.58 to 8.84]	0.5318
Maximal capillary density				
Birth	104.23 (16.39)	117.12 (19.22)	12.83 [8.77 to 16.88]*	<0.0001
3 months	72.76 (11.51)	67.40 (12.15)	–3.70 [–12.01 to 4.61]	0.3809
6 months	57.71 (10.23)	62.78 (10.88)	3.97 [–4.26 to 12.19]	0.3425
12 months	55.33 (11.07)	57.41 (11.77)	1.84 [–5.29 to 8.97]	0.6118
Systolic BP				
Birth	73.77 (10.67)	76.45 (11.38)	2.71 [1.09 to 6.50]	0.1580
3 months	85.47 (15.43)	80.92 (8.45)	–6.02 [–17.17 to 5.14]	0.2833
6 months	88.06 (12.65)	82.00 (18.22)	–7.09 [–18.01 to 3.83]	0.1981
12 months	88.56 (17.11)	88.63 (15.86)	0.18 [–9.98 to 10.34]	0.9717
Diastolic BP				
Birth	46.62 (10.24)	50.77 (10.92)	4.06 [0.46 to 7.67]^†^	0.0281
3 months	49.56 (13.14)	48.33 (16.33)	–2.57 [–13.18 to 8.04]	0.6282
6 months	53.70 (11.28)	48.00 (13.71)	–6.32 [–16.71 to 4.07]	0.2272
12 months	57.91 (16.27)	54.13 (14.98)	–3.60 [–13.17 to 5.97]	0.4531

Between-group differences in BCD and MCD were analyzed using a mixed model in which BW category, measurement time point, and interaction between BW category and measurement time point were treated as fixed effects, and the subject was treated as a random effect. BCD, basal capillary density; CI, confidence intervals; MCD, maximal capillary density; SD, standard deviation

*Significant at *P* < 0.0001.

^†^Significant at *P* < 0.05.

### Preterm and SGA infants

Preterm infants (*n* = 40, 57% of LBW infants) had a higher BCD and MCD at birth when compared to term infants (*n* = 295; MD = 12.06 cap/field, 95% CI [7.42 to 16.69], *P* < 0.0001) and (MD = 8.27 cap/field, 95% CI [–3.08 to 13.47], *P* = 0.002), respectively. Similar to LBW infants, preterm infants underwent a significant capillary remodeling in the 1st 3 months of life (BCD MD = –47.52 cap/field, 95% CI [–37.20, to –57.83], *P* < 0.0001; MCD MD = –46.18 cap/field, 95% CI [–56.62 to –35.74], *P* < 0.0001). After 3 months, there was no significant difference in BCD or MCD between preterm and term infants (Table 4). Similarly, SGA infants (*n* = 80) had a higher BCD and MCD at birth when compared to AGA infants (*n* = 255), (BCD MD = 8.47 cap/field, 95% CI [4.93 to 12.01], *P* < 0.0001; MCD MD = 7.37 cap/field, 95% CI [3.38 to 11.36], *P* = 0.0030). This was followed by a significant reduction in both parameters so that by 3 months, there was no significant difference between the 2 cohorts.

**Table 4.** Mixed model analysis of capillary density at birth, 3, 6, and 12 months in preterm and term infants: between-group comparison

Variable	Term Mean (SD)	Preterm Mean (SD)	Mean difference [95% CI] preterm vs. term	*P* value
Basal capillary density				
Birth	99.01 (15.91)	110.91 (20.45)	12.06 [7.42 to 16.69]*	<0.0001
3 months	69.28 (9.49)	64.44 (11.21)	–5.92 [–16.04 to 4.20]	0.2505
6 months	56.38 (8.28)	61.99 (6.70)	3.22 [–5.01 to 11.45]	0.4414
12 months	50.81 (9.04)	54.65 (10.86)	3.18 [–5.05 to 11.42]	0.4472
Maximal capillary density				
Birth	105.90 (17.19)	114.16 (10.41)	8.27 [–3.08 to 13.47]^†^	0.0020
3 months	72.32 (11.58)	67.67 (12.62)	–4.52 [–14.95 to 5.91]	0.3937
6 months	57.15 (10.21)	67.86 (7.06)	7.58 [–2.00 to 17.15]	0.1203
12 months	55.47 (10.82)	57.93 (13.22)	2.58 [–6.04 to 11.20]	0.5561

Between-group differences in BCD and MCD were analyzed using a mixed model in which prematurity (preterm vs. term), measurement time point, and interaction between term and measurement time point were treated as fixed effects, and the subject was treated as a random effect. BCD, basal capillary density; CI, confidence intervals; MCD, maximal capillary density; SD, standard deviation.

*Significant at *P* < 0.0001

^†^Significant at *P* < 0.01.

### Predictors of CD changes

Non-Caucasian ethnicity predicted a higher BCD (regression coefficient (RC)= –11.32, 95% CI [–17.75 to –4.89], *P* < 0.001) and MCD (RC = –12.20, 95% CI [–19.35 to –5.05], *P* < 0.001) at birth and a more significant percentage reduction in BCD between birth and 3 months (RC = –11.55, 95% CI [–22.85 to –0.25], *P* = 0.0452). A lower gestational age predicted a significantly higher BCD at birth (RC = –2.98, 95% CI [–4.07 to –1.88], *P* < 0.0001), 3 months (RC = –1.93, 95% CI [–3.33 to –0.52], *P* = 0.0078), and 6 months (RC = –1.41, 95% CI [–2.55 to –0.28], *P* = 0.0155) but only predicted a higher MCD at birth (RC = –2.24, 95% CI [–3.46 to –1.03], *P* < 0.001) and 6 months (RC = –2.14, 95% CI [–3.55 to –0.73], *P* < 0.005). All other maternal parameters or predefined measures of infants’ growth (including maternal BMI at booking, antenatal history of gestational diabetes mellitus, family history of hypertension, gestational age of the infant at birth (weeks), maternal age at time of baby’s birth, and smoking history before pregnancy) were not found to be statistically significant predictors of CD changes.

### BP results

At birth, LBW infants had a significantly lower diastolic BP than NBW infants (MD = –4.06 mm Hg, 95% CI [–7.67 to –0.46], *P* = 0.0281; Table 3). Systolic BP in NBW infants increased significantly from birth to 3 months, (MD = 12.23 mm Hg, 95% CI [6.35 to 18.11], *P* = 0.0001) and remained relatively constant thereafter (Table 2). We identified a significant negative correlation between systolic BP and MCD at 3 months for the whole sample (*r* = –0.4762, *P* = 0.0458). Furthermore, the change in CD predicted the change in BP (RC = 0.36, 95% CI [–0.000 to 0.72], *P* = 0.0503. At 12 months, both systolic BP and diastolic BP in the NBW infants were significantly higher compared to levels at birth (MD = 15.22 mm Hg, 95% CI [7.36 to 23.08], *P* = 0.001) and (MD = 13.18 mm Hg, 95% CI [4.02 to 22.33], *P* = 0.008), respectively. BP did not change significantly in the LBW between birth and 12 months.

## DISCUSSION

This study demonstrates that LBW infants born to mothers normotension, irrespective of their gestation at birth (i.e., born at term or preterm), have a significantly higher CD at birth when compared to NBW infants.^10^ LBW infants then undergo an “accelerated capillary remodeling” in the 1st 3 months of postnatal life resulting in the normalization of their higher CD. We also report, for the 1st time, that NBW infants demonstrate a continuous but gradual reduction in their BCD up to 12 months of age, and a similar reduction in MCD up to 6 months of age only. We are not aware of any other studies that have examined the capillary microcirculation in infants with LBW or NBW over a whole year.

Infants have a vast and dense capillary surface area available for fluid, oxygen, solute, and protein exchange, most notably in the peritoneum, muscle, and skin.^19^ Thus, previous studies have suggested that the capillary network in the conjunctiva is structurally extensive and “disorderly” at birth before it matures into a more effective network between 14 and 17 weeks.^20^ It makes teleological sense to accept that our findings in the 1st 3 months of life represent a period of plasticity during which a normal physiological phenomenon of “pruning” or “remodeling” of capillaries is seen in NBW infants. Top et al. ^15^, for instance, examined the capillary microcirculation in the buccal mucosa in term neonates and found higher BCD in infants less than 1 week old compared with older children, confirming that BCD decreases progressively from as early as 1 week following birth. Suichies et al. showed that NBW infants born at term undergo a significant reduction in skin blood flow in the 1st 5 days of life, and they suggested that this is due to morphological and regulatory changes in the microcirculation of the skin.^21^ Alternatively, it could be postulated that this physiological process of remodeling could be initiated antenatally. In keeping with this, LBW infants who are generally born prematurely may not have undergone the same extent of capillary loss as their NBW counterparts born at term. This would explain the higher CD at birth found in LBW infants and in preterm infants. It would also explain our finding that lower gestational age predicts a higher capillary density at birth.

### CR in individuals with a history of LBW

Multiple studies have established that various micro- and macrovascular markers of hypertensive risk such as CR, increased intima–media thickness, arterial stiffness, and endothelial dysfunction are present in children and young adults with a history of LBW.^22^ CR, which is known to increase peripheral vascular resistance and has been confirmed to antedate the onset of hypertension,^2–6^ has specifically been identified in the microvasculature of the retina^14,23^ and the dermal microcirculation of prepubertal children.^12,24,25^ However, very few studies have investigated CR in newborn infants with LBW. Goh et al.^26^ examined skin CD in 17 LBW infants and 21 high BW infants at 1 postnatal time point (3 months of age) and found no difference in BCD between the 2 groups. As CD was only measured once, at 3 months, they were unable to observe the significant accelerated capillary remodeling that we found in our study. Therefore, our new findings provide an explanation for the results of Goh et al.^26^; as we too found no difference between NBW and LBW infants at 3 months and suggest that LBW infants, having undergone an accelerated capillary remodeling, bring their CD to a level equivalent to that of NBW infants. van Elteren et al.^27^ studied the microcirculation in infants and found that both preterm (<32 weeks’ gestation) and term neonates undergo a significant reduction in their total vessel density (TVD) in the 1st month of life and that preterm infants have a consistently higher TVD than term infants.^27^ The authors suggested that this is an adaptation of the cutaneous microcirculation at birth and that differences in antenatal oxygen exposure may explain differences in the way the microcirculation develops in the early postnatal period. However, when we investigated the effects of oxygen therapy on CD and BP in LBW infants in a recent study, we found that oxygen therapy was associated with higher BP levels at 40 weeks postnatally, but had no effect on CR.^28^

Conversely, studies investigating LBW and its association with CR in older children and young adults have provided inconsistent results. For instance, Bonamy et al.^12^ studied 39 school children (aged 7–12 years) with a history of very preterm birth and LBW. They found that their subjects had a significantly lower BCD, but not MCD, when compared with 21 NBW infants born at term.^12^ Similarly, Lewandowski et al.^29^ recently studied young adults (aged 20 –30 years) with a history of preterm birth and LBW and found that these individuals had significant rarefaction of their BCD and MCD, a higher BP, and a relatively exaggerated antiangiogenic state when compared with their term-born counterparts.^29^ Irving et al.^30^ studied young adults (mean age 24 years) with a history of LBW and found no difference in BCD or MCD. It must be stressed here that both studies of Bonamy et al.^12^ and Lewandowski et al.^29^ included individuals born to mothers with hypertensive disorders of pregnancy that has been shown to affect microvascular remodeling through the release of antiangiogenic factors such as soluble endoglin and sFlt-1 into the maternal circulation and potentially into the fetal circulation.^18,31^ Our study is, therefore, unique in that we only included LBW infants born to mothers with normotension to eliminate any effects of maternal hypertension or antiangiogenic factors on the neonatal microcirculation. Recent studies have suggested that preterm birth, more so than LBW, is the predominant factor associated with an adverse vascular profile, but this issue is still debated. However, our results show that LBW infants born at term or preterm show significantly higher CD profile at birth and parallel accelerated capillary remodeling in the 1st 3 months of postnatal life.

Our novel finding that infants of South Asian and Black ethnicity had higher BCD and MCD at birth and a more significant percentage reduction in CD between birth and 3 months than their Caucasian counterparts is very intriguing and requires further investigation. It is well known that Black and South Asian individuals are at a higher risk of cardiovascular morbidity and mortality that cannot be explained by the traditional CVD risk factors.^32^ We and others have previously demonstrated that adult South-Asian individuals with normotension have a reduced BCD and MCD when compared to Caucasian controls with normotension.^33–35^ Larger studies conducted in infancy and throughout life in these individuals are warranted to define the independent effect of ethnicity on the postnatal microcirculation and future CVD risk.

### BP and capillary changes in the 1st year of life

Our finding of an inverse association between systolic BP and MCD at 3 months is novel and suggests that capillary remodeling may induce the rise in BP at 3 months of life. Our finding of a lower diastolic BP in LBW infants confirms the results of Gillman et al.^36^ who published a study on perinatal predictors of newborn BP and showed that LBW is associated with low BP in newborns. Interestingly, Launer et al.^37^ showed a direct association at age 1 week and an inverse association at age 3 months, suggesting that the direction of the LBW and BP association reverses at some point between these 2 ages. We speculate here that the accelerated capillary remodeling we observed in the 1st 3 months of life possibly mediates the reversal of the association between LBW and BP.

## CONCLUSIONS

We have shown that LBW infants born to mothers with normotension have a significantly higher CD at birth compared to NBW infants. NBW infants showed a gradual reduction in CD in the 1st 12 months of life with the greatest reduction occurring between birth and 3 months. More interestingly, LBW infants underwent a process of accelerated capillary remodeling in the 1st 3 months life, by the end of which they had a CD similar to that of NBW infants. We also found a significant negative correlation between systolic BP and MCD at 3 months, which may provide a strong evidence for the role of CR in the causation of hypertension. This process of accelerated capillary remodeling is unlikely to explain the CR observed in adult individuals with a history of LBW. We, therefore, postulate that another process of “capillary hyper-pruning”^10,16^ is likely to occur after the 1st year of life but before adolescence. Identifying the exact timing of CR may provide an opportunity to prevent or reverse these microcirculatory abnormalities and thereby prevent future hypertension.
